# Masculinity, well-being, aggression and aging: Associations with sex hormones in current and past users of anabolic steroids and weightlifting controls

**DOI:** 10.1192/j.eurpsy.2026.10900

**Published:** 2026-07-31

**Authors:** A. Bjørnebekk, M. E. Scarth, P. M. Thorsby, A. M. Kazmierowska

**Affiliations:** 1 Division of Mental Health and Addiction; 2 Department of Medical Chemistry, Oslo University Hospital, Oslo, Norway

## Abstract

**Introduction:**

Strength, vitality, and aggression are central elements of cultural masculinity, often linked to testosterone. Anabolic-androgenic steroid (AAS) use alters circulating sex hormones in both the short and long term, with potential consequences for identity, behavior, and mental health. Yet, the role of sex hormones in men’s health and psychosocial functioning is often oversimplified and few have systematically examined these associations.

**Objectives:**

To investigate associations between AAS use, well-being, symptoms of male aging, masculinity norms, aggression, and sex hormone levels in midlife male weightlifters.

**Methods:**

This ongoing study examines health and well-being in weightlifters with current, past, or no history of AAS use. Participants (mean age ∼45 years) completed questionnaires assessing masculinity norms (Conformity to Masculine Norms Inventory–30), aggression (Buss–Perry Aggression Questionnaire - Short Form), well-being (WHO-5), and aging-related symptoms (Aging Males’ Symptoms scale). Blood samples were analyzed for testosterone (T), free testosterone index (FTI), follicle-stimulating hormone (FSH), sex hormone-binding globulin (SHBG), luteinizing hormone (LH), and estradiol. Based on self-reported history, participants were stratified into four groups: weightlifting controls (WLC, n=44), current AAS users (n=31), past AAS users (n=24), and past AAS users on testosterone replacement therapy (TRT, n=13). Correlations and group comparisons explored associations between hormones, masculinity, aggression, well-being, and aging symptoms.

**Results:**

Preliminary analyses revealed associations between hormonal markers and psychosocial measures, alongside several non-significant findings. Past AAS users reported lower quality of life and more aging symptoms compared to all other groups (p < .01–.05). Groups did not differ significantly on aggression, but all AAS groups scored higher than WLC on masculinity norms (p <.01). Low T and FTI were associated with more aging symptoms, while in WLC, SHBG levels correlated positively with well-being. Several CMNI subscale findings confirmed classical stereotypes: endorsement of casual sexual activity and multiple partners (playboy subscale), power over women, and career pursuit were all positively associated with T, FTI, and estradiol. No relations were seen for aggression and hormonal markers.

**Conclusions:**

Preliminary findings suggest that AAS history and hormonal status are linked to well-being, aging symptoms, and masculine norms in midlife men. Aggression showed no hormonal associations, whereas specific masculinity dimensions did, reflecting cultural stereotypes. The results highlight the complex interplay of biological and psychosocial factors in men’s health and warrant cautious interpretation and further study.

**Disclosure of Interest:**

None Declared

